# A Method for Generation Phage Cocktail with Great Therapeutic Potential

**DOI:** 10.1371/journal.pone.0031698

**Published:** 2012-03-01

**Authors:** Jingmin Gu, Xiaohe Liu, Yue Li, Wenyu Han, Liancheng Lei, Yongjun Yang, Honglei Zhao, Yu Gao, Jun Song, Rong Lu, Changjiang Sun, Xin Feng

**Affiliations:** College of Animal Science and Veterinary Medicine, Jilin University, Changchun, People's Republic of China; Los Angeles Biomedical Research Institute, United States of America

## Abstract

**Background:**

Bacteriophage could be an alternative to conventional antibiotic therapy against multidrug-resistant bacteria. However, the emergence of resistant variants after phage treatment limited its therapeutic application.

**Methodology/Principal Findings:**

In this study, an approach, named “Step-by-Step” (SBS), has been established. This method takes advantage of the occurrence of phage-resistant bacteria variants and ensures that phages lytic for wild-type strain and its phage-resistant variants are selected. A phage cocktail lytic for *Klebsiella pneumoniae* was established by the SBS method. This phage cocktail consisted of three phages (GH-K1, GH-K2 and GH-K3) which have different but overlapping host strains. Several phage-resistant variants of *Klebsiella pneumoniae* were isolated after different phages treatments. The virulence of these variants was much weaker [minimal lethal doses (MLD)>1.3×10^9^ cfu/mouse] than that of wild-type K7 countpart (MLD = 2.5×10^3^ cfu/mouse). Compared with any single phage, the phage cocktail significantly reduced the mutation frequency of *Klebsiella pneumoniae* and effectively rescued *Klebsiella pneumoniae* bacteremia in a murine K7 strain challenge model. The minimal protective dose (MPD) of the phage cocktail which was sufficient to protect bacteremic mice from lethal K7 infection was only 3.0×10^4^ pfu, significantly smaller (p<0.01) than that of single monophage. Moreover, a delayed administration of this phage cocktail was still effective in protection against K7 challenge.

**Conclusions/Significance:**

Our data showed that the phage cocktail was more effective in reducing bacterial mutation frequency and in the rescue of murine bacteremia than monophage suggesting that phage cocktail established by SBS method has great therapeutic potential for multidrug-resistant bacteria infection.

## Introduction

Lytic bacteriophages are viruses that infect bacteria, hijack their machinery, replicate intracellularly and are finally released by host cell lysis [1]. From the early 1920s, phage therapy has been considered as antimicrobial agents for the treatment of bacterial infectious diseases. However, the development of this therapy has been hampered by the advent of antibiotics [2]. Due to the emergence of multidrug-resistant bacteria, phage therapy has been resurrected during the past few decades [2], [3], [4]. Phage therapy might be a viable alternative to or complement conventional antibiotic therapy because it has already been proven to be advantageous as these are very specific, accurate and potent than antibiotics [5], [6], [7]. Another advantage of using phages over antibiotics is that phages can replicate at the site of infection and thus become available in abundance at the desired site [8]. In addition, several recent and well-controlled animal studies have demonstrated the potential of phages for antibacterial therapy [9], [10], [11].

*Klebsiella pneumoniae* is an opportunistic pathogen frequently associated with urinary tract, bloodstream and intra-abdominal infections, pneumonia, and bacteremia in hospitalized persons whose immunity is compromised by underlying diseases [12], [13]. Bacteremia caused by *K. pneumoniae* usually leads to significant morbidity and mortality in the general population [14]. Treatment of these infections has become ever more difficult due to the prevalent of multidrug-resistant strains [15], [16]. Hence, there is urgency to explore new therapeutic options.

Virulent phages specific to *K. pneumoniae* cells have been studied to control the infection caused by this pathogen [15], [17], [18]. However, the fast emergence of resistant variants during phage treatment is one of the most serious problems [19], [20]. Previous studies have indicated that the phage cocktail can delay the appearance of phage-resistant variants and enhance treatment efficacy [21], [22]. In the present study, we established a “Step-by-Step” (SBS) approach to isolate different phages using wild-type isolates and consecutively phage-resistant variants as host bacteria. The therapeutic potential of the phage cocktail based on this method was further examined in murine model of infection.

## Results

### The morphology and host range of bacteriophages

**Figure 1:**
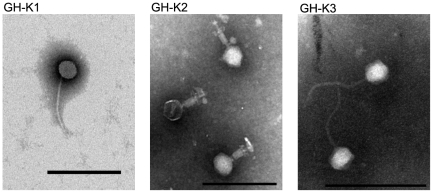
Morphology of phages as revealed by TEM. Three phages displayed different morphology. GH-K2 displayed a short and contractile tail. The tails of GH-K1 and GH-K3 were long, and the tail of GH-K3 appeared more flexible than that of GH-K1. The bars represent 200 nm.

GH-K1, GH-K2 and GH-K3 were isolated using K7, K7R^1^ and K7R^2^ as host strains respectively by the SBS method. These phages were examined under the Transmission Electron Microscope and showed significant morphological variation (Fig. 1). GH-K1 and GH-K3 appeared to be similar in morphology whereas GH-K2 looked different. GH-K2 had an isometrically hexagonal head of 60±5 nm in diameter, a contractile tail measuring 40×20 nm, and a double layer baseplate of about 30 nm in diameter, to which 20-nm short tail spikes were attached. The head was separated from the tail sheath by a collar. The observed morphology suggested that GH-K2 was a member of the *Myovirus* family. GH-K1 displayed an isometrically hexagonal head of 40±5 nm in diameter and a long tail of 180±5 nm. GH-K3 had an isometrically hexagonal head of about 50 nm in diameter and a 150-nm-long tail. In addition, the tail of GH-K3 appeared to be more flexible compared with that of GH-K1. All phages formed clear plaques in the early stage when cultured with K7 strain, and several hours later, the plaques became larger and were surrounded by a large halo. The phage cocktail used in this study was composed of these three phages.

The bacterial host range of three phages was further investigated. All phages displayed lytic activity against K7 strain. In addition to lyse the specific host strain, these phages were able to lyse many other *K. pneumoniae* strains These phages showed a broad host range and the host spectra were different as presented in Table 1. More importantly, of the 34 remaining *K. pneumoniae* samples tested, the combination of three phages was lytic against 30 strains. It's also worth to mention that GH-K2 displayed lytic ability against BAA-2146, a New Delhi metallo-β-lactamase 1 (NDM-1) producing strains. However, no lytic activity against *Streptococcus pneumoniae*, *Bacillus subtilis*, *Salmonella enteritidis*, *Staphylococcus aureus*, or *Escherichia coli* was detected.

**Table 1:** Susceptibility of strains to different phages.

Organism	Strain	Source^a^	Phages		
			GH-K1	GH-K2	GH-K3
*K. pneumoniae*	K7	1	•	•	•
	K7R^1^	1	○	•	○
	K7R^2^	1	•	○	•
	K7R^3^	1	○	•	○
	K7R^123^	1	○	○	○
	BAA-2146	2	○	•	○
	K01-K34	1	15N/19○	21N/13○	12N/22○
*Streptococcus pneumoniae*	CVCC606	3	○	○	○
*B. subtilis*	EA751	1	○	○	○
*S. enteritidis*	CVCC541	3	○	○	○
*E. coli*	ATCC 25922	2	○	○	○
*S. aureus*	ATCC 25923	2	○	○	○

a 1: isolated from the First Hospital of Jilin University; 2: laboratory collection; 3: purchased from China Institute of Veterinary Drug Control; 4: purchased from American Type Culture Collection.

### Characterization of K7 and variants

**Figure 2:**
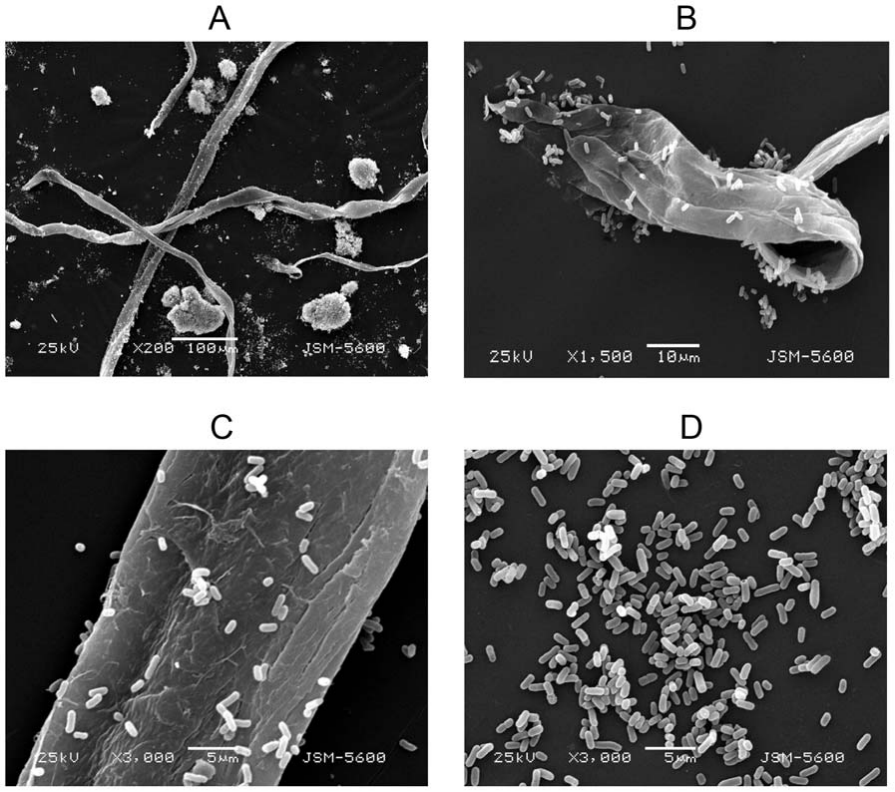
Electron micrographs of *K. pneumoniae* strains. A, B, and C show K7, and D shows K7R^1^. B and C are higher magnification views of A. C and D are at the same magnification. The images show that K7 adsorbed to the thick jelly and formed large bacterial aggregates.

Phage-resistant strains were isolated from different bacterial cultures in the present of phage(s) [K7R^1^ from K7 cultures in the presence of GH-K1; K7R^2^ from K7R^1^ cultures in the presence of GH-K2; K7R^3^ from K7R^2^ cultures in the presence of GH-K3; K7R^123^ from K7 cultures in the presence of GH-K1, GH-K2 and GH-K3]. Compared with K7, which formed large smooth colonies, all variants formed small rough colonies. Variant strains displayed reduced growth rate during the exponential growth phase. The bacterial growth was monitored by measuring culture turbidity (OD_600_). After incubation for 3 h, broth cultures of the variant strains had an OD_600_ of ≤0.4 compared with that of K7 (OD_600_ reached 0.65 at the same condition). In addition, the broth culture of K7 was so viscid that it's difficult to precipitate by centrifugation. Although the morphology of single bacteria did not differ among the different strains under scanning electron microscopy (SEM), most K7 cells were in the form of large bacterial aggregates and some adhered to the thick jelly (Fig. 2), while the variants scatterly located. This morphology feature of variant strains remained stable even after repeated subculture and storage at −70°C.

There was significant disparity in the MLD between K7 and variants. The variants displayed much weaker virulence, with a MLD>1.3×10^9^ cfu. In contrast, the MLD of K7 was only 2.5×10^3^ cfu. In addition, when 10^8^ cfu variants was intraperitoneally injected into mice, the bacteria counts declined quickly and became undetectable in mice 7 days after injection, and the infected mice didn't show any signs of physical stress (ruffled fur or general lethargy).

### The mutation frequency was reduced by phage cocktail

When K7 were cocultured with different phage(s), there was an increase in OD_600_ at the beginning. After coculture for 1–2 h, lytic disintegration of cells resulted in a conspicuous decrease of OD_600_. The phage cocktail induced a sharp decrease of OD_600_ from 0.5 to 0.06 within 1.5 h, while monophage treatment resulted in a much slower decrease of OD_600_ from 0.65±0.05 to 0.12±0.04 within 2.5 h. Otherwise, normal bacterial culture in the absence of phage showed a consistent increase in OD_600_ examined at different time points. It took 6–8 h for monophage-treated bacteria or 26 h for phage cocktail-treated bacteria to regain apparent growth. The production of resistant variant following phage cocktail treatment (cfu/mL ± SD, 7.5±0.3×10^−7^) was significantly less (p<0.01) compared with monophage treatment (cfu/mL ± SD, GH-K1 = 9.5±0.23×10^−4^, GH-K2 = 3±0.36×10^−5^, GH-K3 = 4±0.2×10^−4^).

### Phage treatment of systemic infections

To produce a murine bacteremia model, a heavy dose of K7 was administrated intraperitoneally. An acute bacteremia was induced at a challenge dose of 2.5×10^8^ cfu per mouse. This murice model was used for the phage therapy studies. Bacteremia was detected after 30 min after K7 challenge. The bacterial count in the blood reached 3.6×10^6^ cfu/mL. At this point, the bacterial counts in heart, liver and spleen were more than 2.3×10^5^ and the infection was systemic.

**Figure 3:**
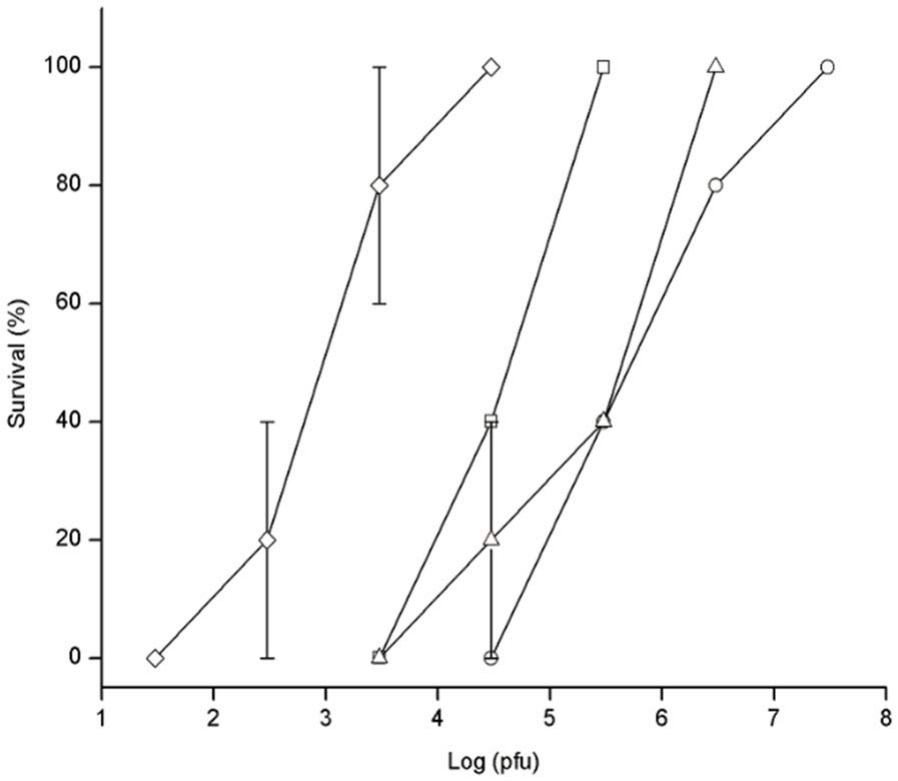
Survival rate of bacteremic mice treated with different doses of phages. Mice were inoculated intraperitoneally with K7 at the dose of 2.5×10^8^ cfu. Thirty minutes later, different doses of GH-K1 (○), GH-K2 (□), GH-K3 (Δ), or phage cocktail (◊) were injected into the peritoneal cavity of the mice. Every group contained five mice and each symbol represents the average of three experiments. The error bars indicate SD.

**Figure 4:**
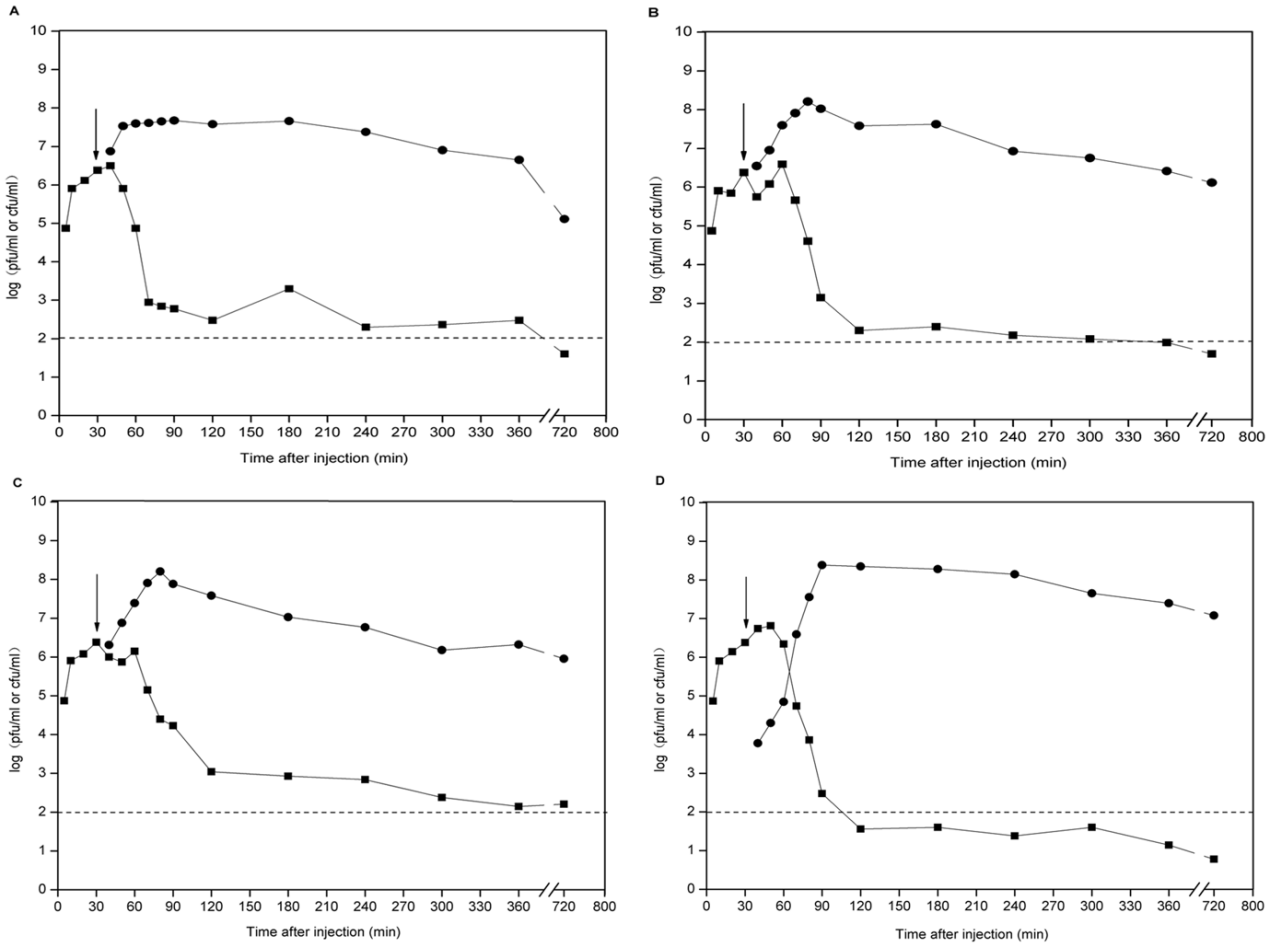
Colony counts of bacteria and titers of phages in blood samples obtained at regular intervals. The mice were challenged with K7 at the dose of 2.5×10^8^ cfu. At the indicated times, bacterial counts (•) and phage titers (▪) in three mice treated with either the MPD of (A)GH-K1, (B)GH-K2, (C)GH-K3, or (D) the phage cocktail were determined from peripheral blood samples taken from the caudal vein. The arrow indicates the moment at which the phage was injected (30 min after challenge). Each symbol represents the average of three experiments.

**Figure 5:**
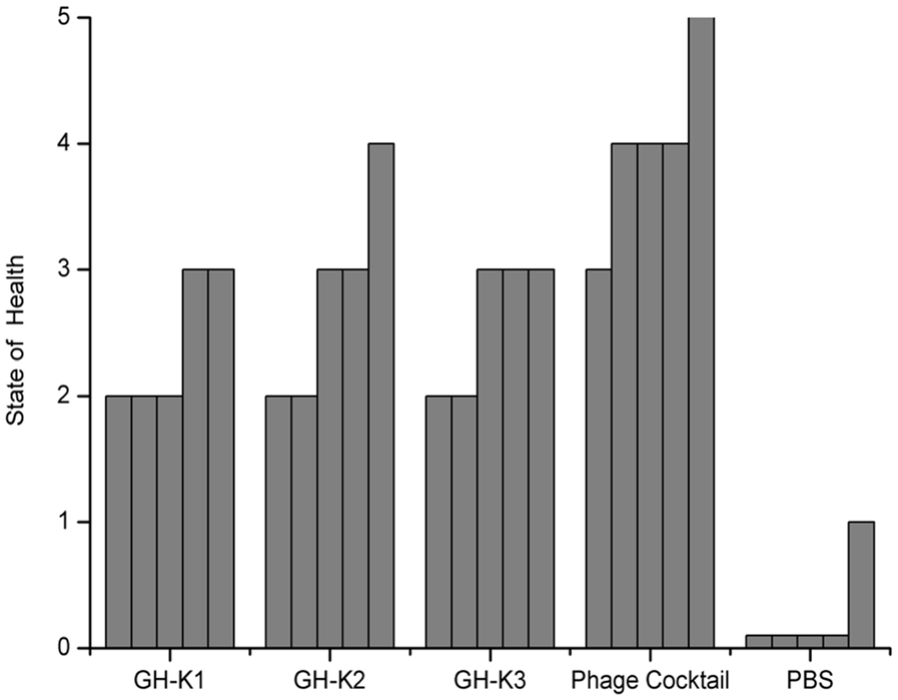
Comparison of the mice states at 12 h after treated with different single phage and the phage cocktail. Four groups of five mice were treated with different single phage and phage cocktail at the MPD, while the control group was treated with PBS. The mice were scored for their state of health on a scale of 5 to 0, based on progressive disease states. A score of 5 indicates normal health and unremarkable condition. Slight illness, defined as decreased physical activity and ruffled fur, was scored as 4. Moderate illness, defined as lethargy and a hunched back, was scored as 3. Severe illness, with the aforementioned signs, plus exudative accumulation around partially closed eyes, was scored as 2. A moribund state was scored as 1. Death was scored as 0. Each bar indicates the state of health of a single BALB/c mouse.

Different monophage and phage cocktail were injected intraperitoneally 30 min later after K7 challenge. As shown in Fig. 3, the MPDs of GH-K1, GH-K2, GH-K3, and cocktail were 3.0×10^7^, 3.0×10^5^, 3.0×10^6^, and 3.0×10^4^ pfu/mouse, respectively. The bacterial counts in the blood decreased to 3.0×10^2^, 2.0×10^2^, and 1.1×10^3^ cfu/mL 2 h after treatment with GH-K1, GH-K2, and GH-K3, respectively (Fig. 4). Importantly, bacteremia dropped significantly after 2 h treatment with phage cocktail, reaching 3.6×10^1^ cfu/mL. The phage cocktail treated-mice were more healthy than any monophage-treated mice (Fig. 5).

When a single injection of GH-K1, GH-K2, or GH-K3 at 3.0×10^4^ pfu/mouse was administered, the mice survival rates were 0%, 40%, and 20%, respectively. The phage cocktail significantly enhanced the protection of bacteremic mice against lethal K7 infection (*P*<0.01). Even if the dose was reduced to 3.0×10^3^ pfu, the phage cocktail sufficiently protected four out of the five mice tested. As controls, the heat-inactivated phage cocktail and buffer treatment were ineffective. Moreover, the administration of a single excess dose of phage cocktail (3.0×10^9^ pfu) did not produce any adverse effects (fever or general lethargy) 30 days after injection.

### Effect of delay in treatment

**Figure 6:**
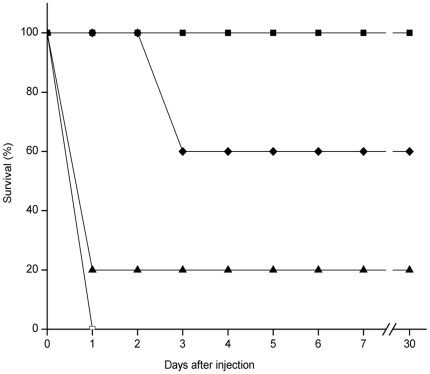
Delayed treatment with phage cocktail. Mice were inoculated intraperitoneally with K7 at the dose of 2.5×10^8^ cfu. Cocktail phages at the dose of 3.0×10^4^ pfu or a buffer were administered into the peritoneal cavities of mice at the indicated time intervals after challenging with K7. Phage cocktail was given at 1 h (black squares), 2 h (black diamond), or 3 h (black triangles) after the K7 challenge. Infected mice treated with buffer (white squares) under the same conditions were used as control. Each symbol represents the average of three experiments.

A life-saving effect was also observed even when the phage cocktail at the MPD was administered at 1 h and 2 h after K7 challenge. The mice model became generally lethargic and hunch-backed at these time points. The protective rate also reached 100% and 60% respectively (Fig. 6). Even when administered at 3 h, the phage cocktail also displayed effective protection (100%) after increasing the dose from 3.0×10^4^ pfu to 6.0×10^6^ pfu.

## Discussion

Bacteriophage could be an alternative to antibiotics for the treatment of multidrug-resistant bacterial infections. Indeed, it has proven to be medically superior to antibiotic therapy in many ways [23]. However, the emergence of phage-resistant variants was observed rapidly if only one phage strain was used against a particular bacterium [22]. Fortunately, there are an abundance of other phage species which possess lytic ability against resistance-obtained variants [5].

In the present study, we report a SBS method. The method takes advantage of the occurrence of phage-resistant variants and ensures phages lytic for wild-type strain and its phage-resistant variants are selected. The phage cocktail established by this method can not only display broad lytic range, but also ensure that bacteria resistant to one phage remain susceptible to others. Hence, the cocktail established by SBS is significantly different from previous phage cocktails [24], [25].

Three phages have been isolated using the SBS method in this study. If cells which resistant to phage “X” are sensitive to phage “Y,” it can be concluded that the adsorption receptors or the infection mechanism of phage “X” and “Y” are different [26], [27], [28]. In this sense, GH-K1, GH-K2 and GH-K3 could identify different receptors or possess different infection mechanism.

The avirulence and reduced persistence of these variants in vivo were in agreement with previous studies [29], [30]. Perhaps, the occurrence of this phenomenon was because the variants could be cleared rapidly by innate immunity [31]. Otherwise, in the case of *K. pneumoniae*, the virulence is related to the surface-specific polysaccharide antigen (SPA), which acts as an initial surface binder that helps the organisms gather together or form biofilms [15]. Maybe, compared with K7, SPA on the variant strains might have been changed or missed.

In most cases, the appearance of phage-resistant mutants was not considered important as earlier studies have reported that resistant variants tended to be avirulent and were easily taken care by phagocytes and immune system [29], [30]. However, the fast appearance of phage-resistant variants might limit the application of phage therapy. From the experiment in vitro, it was demonstrated that the phage cocktail significantly reduced the frequency of mutation compared with any monophage, and displayed higher lytic efficacy. Although the resistant variants also appeared when the cocktail was used, it needed a much longer time to accumulate enough mutations to develop resistance to three phages [28]. In addition, the long-term inhibition function was sufficient for the immune system of mice to eliminate the small number of avirulent variants.

More importantly, compared with any single phage, the phage cocktail effectively rescued *Klebsiella pneumoniae* bacteremia in the murine K7 strain challenge model. In addition, the dynamics of bacteria in the blood demonstrated that the cocktail needed a smaller dose, but displayed stronger elimination of bacteria. Maybe, it can be concluded that the phage cocktail proliferation threshold was higher than that of monophage in vivo [32], [33]. Since, a small amount of phage cocktail can proliferate quickly, resulted in the fast elimination of bacteria in infected mice and lower the development of phage resistance. Coupled with the data obtained from the experiment in vitro, it showed that these three phages might have a synergistic effect on killing K7, at least based on these limited studies.

In our opinion, it will be more reasonable to design corresponding phage cocktail according to the specified pathogen. A large number of phages need be isolated and identified and the phage library (warehouse) should be established. The phage cocktail could be formed using SBS method from the library or medical institution and prepared in advance.

Overall, the data presented in this study showed that phage cocktail established by SBS method has great therapeutic potential for multidrug-resistant bacteria infection.

## Materials and Methods

### Animals

All animal studies were conducted according to the experimental practices and standards approved by the Animal Welfare and Research Ethics Committee at Jilin University (Approval ID: 20110520-3). Animal experiments were carried out on 20∼22 g female BALB/c mice.

### Bacterial strains and culture conditions

K7 was *K. pneumoniae* strain which was isolated from a clinical specimen (obtained from a patient in the People's Hospital of Jilin Province, China) and showed a very strong virulence. Antimicrobial susceptibility tests revealed that K7 was resistant to most of the commonly used drugs. K7R^1^, K7R^2^, K7R^3^ and K7R^123^ were phage-resistant variants which were isolated from K7 cultures treated by phage(s) according to the method of Verma et al. [15]. Bacteria that grew after phage treatment were isolated and re-subjected to the same phage(s). Single bacterial colonies were selected randomly from the plate post-incubation and biochemically verified to be *K. pneumoniae*. *Staphylococcus aureus* and *Streptococcus pneumoniae* were cultured in brain heart infusion (BHI) broth, while other strains were routinely cultured in the Luria-Bertani (LB) medium with constant shaking (200 rpm) at 37°C for 16–18 h.

### Step-by-Step (SBS) method

Step-by-Step (SBS) was an approach which isolated phages using wild-type bacteria and consecutively phage-resistant variants as hosts. Simply, the first and the second phages were isolated using wild-type bacteria and the first phage-resistant variant (resistant to the first phage) as hosts, respectively. The next variant, resistant to the second phages, was used to isolate the third phage. The rest could be deduced by analogy. The process of phage isolation was stopped until the last phage-resistant bacteria were sensitive to forward isolated phage. The cocktail comprised an equal amount of each phage.

### Phage isolation, purification, and host range

Bacteriophages active against *K. pneumoniae* were isolated by using samples obtained from a Changchun sewage treatment plant by the SBS approach. The purification of phages was achieved according to the conventional method [34]. Different bacteriophages were isolated and stored in 50% glycerol at −70°C. Purified phage samples in SM buffer [100 mM NaCl, 8 mM MgSO_4_, 50 mM Tris-HCl (pH 7.5), 0.01% gelatin] were negatively stained with phosphotungstic acid (2% w/v) and examined by transmission electron microscopy (Hitachi Co. Ltd., Tokyo, Japan) at an accelerating voltage of 80 kV. To detect the host range of phages, the spot test was carried out as described previously [35].

### Comparison of K7 versus its variants

K7 and its variants were cultured on LB agar and the colony morphology was observed. Cultures of K7 and its variants grown to mid-exponential phase were harvested and washed twice with PBS. Samples of these strains were visualized at 25 kV using a JSM-5600 scanning electron microscope (JEOL Ltd., Tokyo, Japan), after gold sputtering the specimens using a Jeol Fine Coat JFC-1100 ion sputter coater.

Groups of five mice were injected intraperitoneally with different bacterial inoculate to determine the minimal lethal dose (MLD) of every strain that produces 100% mortality over a 7-day follow-up period, as described previously [34].

### The frequency of K7 mutation treated with phage(s)

Different monophage or the cocktail was added to the K7 culture at value of multiplicity of infection equal one when the OD_600_ value reached 0.1. The frequency of phage resistant variants isolation was determined as described previously [9]. Simply, cultured bacteria in liquid exposed to phage(s) were spread plated on agar plates and the colony count was performed after incubation at 37°C overnight. The frequency of mutation was calculated as the number of resistant colonies/mL.

### Ability of phages to protect mice against lethal K7 infection

A heavy dose of K7 (2.5×10^8^ cfu) was injected intraperitoneally into groups of five mice to establish an acute bacteremic model. To detect the state of bacteremia, the bacterial content in internal organs and blood was observed. When the injected bacteria effectively entered into the circulatory system, the minimal protective dose (MPD) of single monophage and cocktail treatment, which achieves a 100% protection rate in bacteremic mice, was determined. Peripheral blood samples were taken from the caudal vein and used for counting of bacterial content (cfu/mL) and phage titer (pfu/mL) as described previously [33], [36]. In addition, the health level of infected mice with bacteria were monitored and scored for the following manifestations indicating progressive disease states: decreased physical activity and ruffled fur, general lethargy and hunch-back posture, exudative accumulation around partially closed eyes, moribund, and death.

### Delayed-treatment experiment

To test the effect of delayed treatment on the ability of phage cocktail to rescue the murine bacteremic model, an additional experiment was performed. A single injection of phage cocktail was administered to groups of five mice at different delayed time points after K7 challenge. Protective ability was evaluated by the protective rate.

### Statistical analysis

SPSS version 13.0 (SPSS, Inc., Chicago, IL, USA) was used for all statistical analyses. Survival rates of mice treated with phages were analyzed using the Fisher exact test. The mutation rate was analyzed by one-way analysis of variance. P<0.01 was considered significant.
